# Detecting Touch-Induced Calcium Dynamics With Live-Cell Imaging in *Torenia* Stigma

**DOI:** 10.21769/BioProtoc.5683

**Published:** 2026-05-05

**Authors:** Xuan Zhou, Xiaofang Ma, Shuibo Yang, Shihao Su

**Affiliations:** State Key Laboratory of Biocontrol and Guangdong Provincial Key Laboratory of Plant Stress Biology, School of Agriculture and Biotechnology, Shenzhen Campus of Sun Yat-sen University, Sun Yat-sen University, Shenzhen, China

**Keywords:** Calcium, Touch, Live-cell imaging, Stigma, *Torenia*

## Abstract

Calcium ions serve as a universal secondary messenger, integrating diverse external signals, such as light, herbivory, and mechanical stimuli, within plant cells. However, the visualization and mechanistic dissection of calcium signaling specifically in response to mechanical stimulation remain technically challenging and underexplored in most plants. Previous studies have been largely confined to a few model systems, including *Arabidopsis*; here, we introduce a live-cell imaging approach using the stigmas of *Torenia fournieri*. This in vitro system enables multiscale observation of calcium signal patterns following controlled mechanical stimulation. This versatile platform not only simplifies the design of calcium imaging assays but also provides a tractable system for functionally validating other key molecular components in this signaling pathway.

Key features

• Live-cell imaging is employed to monitor calcium signals in response to mechanical stimulation, enabling examination at both the whole-organism and cellular levels.

• Stigma vitality is maintained under controlled in vitro conditions throughout the imaging.

## Graphical overview


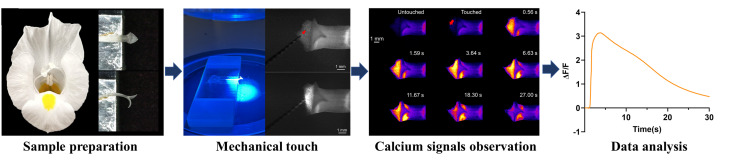


## Background

Plants constantly perceive mechanical stimuli throughout their life cycle, from wind-blown leaves and probing roots to pollen hydration and seed germination. Indeed, a plant's existence can be viewed as a continuous history of responding to such mechanical cues. Upon stimulation, plants upregulate touch-related *TOUCH/TCH* genes, which mediate contact-induced morphogenesis by integrating hormonal pathways involving auxin, cytokinin, ethylene, and jasmonic acid [1,2]. Beyond these slower morphological changes, some plants exhibit rapid movements in response to touch. Leaves of species like the Venus flytrap and *Mimosa pudica* execute rapid movements for insect capture or herbivory avoidance, respectively [3,4]. Thus, external mechanical forces fundamentally shape a plant's adaptive strategies.

Calcium ions (Ca^2+^) serve as one of the most important second messengers within plant cells. Advances in genetically encoded fluorescent indicators now allow real-time observation of Ca^2+^ dynamics in living tissues. Studies utilizing these tools have demonstrated that diverse stimuli trigger pronounced fluctuations in cytosolic Ca^2+^ ([Ca^2+^]_cyt_) concentrations [5–7]. Mechanical stimuli are particularly effective inducers of such Ca^2+^ signals. In *Arabidopsis*, touching the leaf tip generates a calcium signal at the site of contact, which subsequently spreads through the vascular bundles to the petiole [5]. When a single trichome is touched, the resulting calcium signal radiates outward from the trichome as a center [6]. In the touch-sensitive plant *Mimosa pudica*, touching causes the leaflets to close within seconds. During this process, [Ca^2+^]_cyt_ levels rise from the veins to the pulvinus, significantly amplifying in that location [4]. Shortly thereafter, the signal transmits hierarchically to the next pulvini, ultimately causing the entire compound leaf to fold [4]. Notably, the speed of calcium signal transmission in *Mimosa* is much faster than in *Arabidopsis* leaves. Similarly, in the Venus flytrap, after the first touch, [Ca^2+^]_cyt_ concentration rises starting at the touched trigger hair and spreads in the leaf [3]. Following a second touch, the [Ca^2+^]_cyt_ concentration reaches the threshold to trigger closure [3]. Interestingly, this signal remains confined to the stimulated trap and does not propagate via the vasculature or to adjacent leaves [3]. These findings establish that mechanical touch directly initiates Ca^2+^ fluctuation and propagation, which is essential for activating downstream stress-response genes or driving rapid cellular deformations.

Although different plants employ Ca^2+^ as a messenger to transduce external mechanical cues into internal responses, the specific pathways and transduction mechanisms exhibit notable variation. Deciphering the Ca^2+^ signatures elicited by touch across different species is therefore key to understanding the conservation and specificity within this critical signaling network. Here, we have identified the touch-responsive behavior of stigmas in *Torenia fournieri*, a classical model in the study of plant reproduction [9,10]. The bilobed stigma of *T. fournieri* closes within seconds after mechanical stimulation, and we have demonstrated that the generation and propagation of touch-induced Ca^2+^ signals are essential for this stigma movement [11]. In this study, we present an integrated experimental protocol that details how to trigger stigma movement through controlled mechanical stimulation and how to visualize the associated [Ca^2+^]_cyt_ signal on living stigmas during this rapid response. Given that this protocol allows stimulation of a single papilla cell on the inner epidermal surface of the *Torenia fournieri* stigma, it may offer a valuable reference for mechanical stimulation of single-cell structures in plants, including leaf trichomes of *Arabidopsis*.

## Materials and reagents

**Biological materials**

1. Transgenic *T. fournieri* plants expressing the GCaMP6f-NES probe (UBIQUITIN 10 promoter) [12] produce flowers with open stigmas capable of normal movement; this probe can be used by other groups

**Reagents**

1. Distilled water (Sangon Biotech, catalog number: E607017-0500)

2. Absolute ethanol (Sangon Biotech, catalog number: A500737-0005)

**Laboratory supplies**

1. PCR tubes (200 μL) (Sangon Biotech, catalog number: F611542-0001)

2. Scissors (Sangon Biotech, catalog number: F519231-0001)

3. Microsurgical tissue forceps (Vetus, catalog number: SS-JP)

4. Sharp-point tungsten needles (ETRA, model: 0.1 mm × 25 mm)

5. Double-sided tape (3M, catalog number: 55236)

6. Single-sided tape (Scotch, catalog number: 810-CQ33)

7. Microscope slide (25 mm × 75 mm, 1–1.2 mm) (Citotest, catalog number: 10127105P-G)

## Equipment

1. Fluorescence stereomicroscope (Olympus, model: SZX16) equipped with a 1× objective lens (Olympus, model: SDFPLAPO1XPF) and a sCMOS camera (Olympus, model: DP74)

## Software and datasets

1. CellSens Dimension (Olympus, V4.4.1)

2. GraphPad Prism (GraphPad Software, V10.0)

## Procedure

**A. Sample preparation**

**Figure 1.**
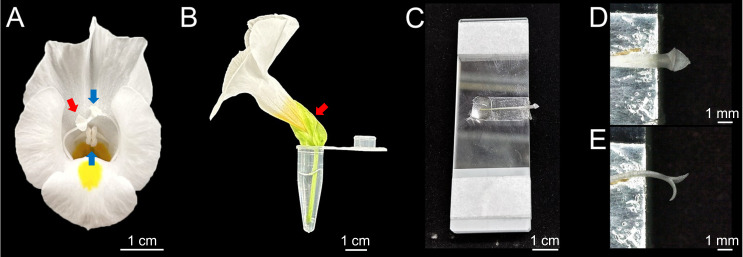
Sample platform setup. (A) Front view of a flower with a motile stigma. Blue arrows indicate the two pairs of anthers; the red arrow indicates the stigma. (B) In vitro culture setup: a flower peduncle inserted into a water-filled centrifuge tube. The red arrow marks the puncture site for isolating the stigma and style. (C) Application of double-sided tape to the center of the sample platform. (D) Upright mounting of the style and stigma, with the inner surface of the upper lobe facing upward. (E) Side mounting of the style and stigma on the platform.

1. Select flowers with stigmas that retain normal motility. Stigmas that have not yet opened will not exhibit observable closure upon touch, while aged stigmas show markedly reduced movement. The optimal stage is when the two pairs of anthers have not yet turned yellow (Figure 1A).

2. Prepare ten 200 μL PCR centrifuge tubes, each containing 150 μL of distilled water.

3. Using sharp scissors, cut the pedicel tip of each selected flower. Immediately insert the cut end into a water-filled tube (Figure 1B). Prepare 10 flowers this way. Under these in vitro conditions, the excised stigmas remain suitable for calcium imaging for 12 h under 25–30 °C, 40%–80% relative humidity, and 300–500 lx.

**B. Stigma-mounting platform setup**

1. Stack five microscope slides longitudinally. Secure the stack at both ends with single-sided tape to prevent sliding.

2. Apply a strip of double-sided tape (20 cm × 1 cm) to the center of the top slide. Peel off one side of the tape to create an adhesive mounting surface (Figure 1C). This platform stabilizes the stigma during manipulation, preventing it from drifting out of the microscope field of view. The stack height provides necessary vertical clearance for stigma movement after mechanical stimulation.

**C. Sample mounting**

1. Extract an in vitro–cultured flower and hold the pedicel. Using forceps, insert the tips approximately 1 cm below the sepal apex and swiftly pinch off the style (Figure 1B; Video 1).

**Video 1.**
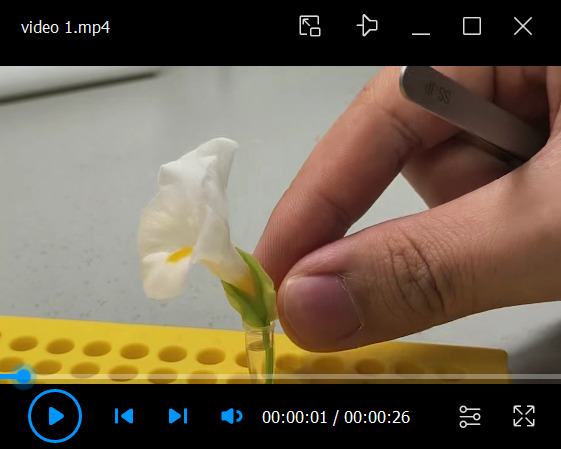
Separation of the style and stigma from the flower

2. Gently grip the style with forceps and press it onto the double-sided tape, orienting the inner surface of the stigma lobes upward. Ensure the junction between the stigma lobes and style extends approximately 5 mm beyond the platform edge. Apply 100 μL of water to the wound of the style on the tap surface to make the style and stigma alive (Figure 1C). The stigma can be mounted either upright or on its side to observe the whole lobe or the marginal papilla cells (Figure 1D, E). The sample is now ready for stimulation and observation.

**D. Mechanical stimulation and imaging of an entire stigma lobe**

**Figure 2.**
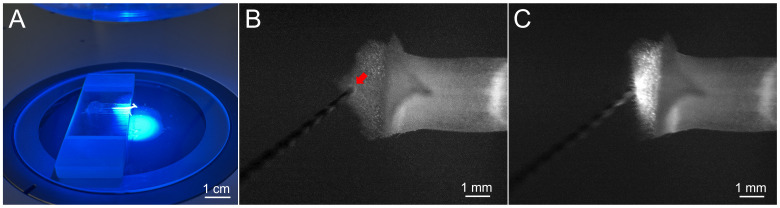
Observation of calcium signals in the stigma of *T. fournieri*. (A) Imaging setup under 488 nm excitation. (B) Initial contact of the needle tip (indicated by the red arrow) with the inner surface of the lobe. (C) Subsequent movement of the lobe induced by mechanical stimulation.

**Figure 3.**
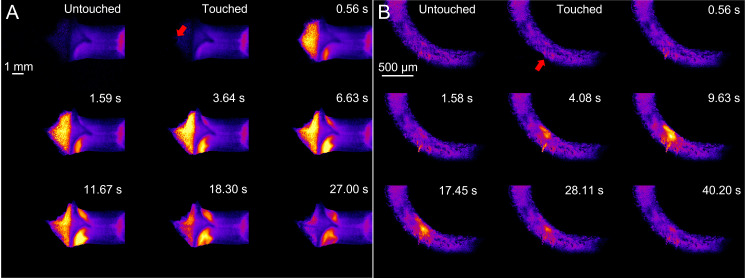
Propagation of touch-induced [Ca^2+^]_cyt_ wave. (A) [Ca^2+^]_cyt_ pattern across the entire lobe after mechanical stimulation (touched region indicated by the red arrow). (B) [Ca^2+^]_cyt_ pattern in a single papillar cell following stimulation (stimulated cell indicated by the red arrow).

1. Position the platform under the microscope objective (Figure 2A). Using 20× magnification, focus on the inner surface of the upper lobe in brightfield. Illuminate the sample with a 130 W, 488 nm excitation source and adjust exposure to 80 ms, gain to 8×, and other parameters until the outline of the lobe becomes clearly visible in the fluorescence channel. The frame rate and imaging duration will automatically adjust following the adjustment of the aforementioned parameters.

2. Clean a tungsten needle with ethanol and initiate recording. Position the needle tip near, but not touching, the stigma within the field of view. Maintaining this orientation, advance the needle at a ~45° angle relative to the lobe's inner surface, briefly touch the stigma until it bends approximately 70° (Figure 2B, C), and then rapidly retract the needle along its axis (Video 2); the needle is kept on the stigma for no more than 500 ms. The resulting [Ca^2+^]_cyt_ signals across the lobe are shown in Figure 3A. After recording, use forceps to remove the style and stigma from the platform before proceeding to the next sample.

**Video 2.**
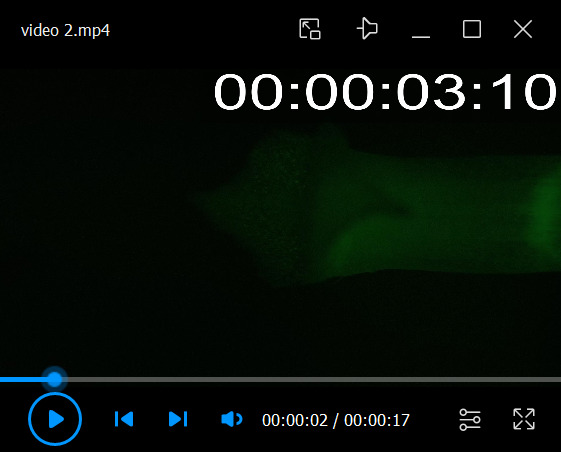
Front view of a stigma with a [Ca^2+^]_cyt_ wave after touch

**E. Mechanical stimulation and imaging of a single papilla cell**

1. For imaging individual papilla cells, mount the style on its side (Figure 1E). The papilla cells at the lobe margin are less densely packed than in the center, facilitating single-cell resolution. Using 50× magnification, locate and focus on a clearly visible marginal papilla cell. Adjust imaging parameters to visualize the cell with minimal background fluorescence.

2. Clean the tungsten needle with ethanol and begin recording. Position the needle tip near the target papilla cell. Gently advance the needle to make brief contact with the cell apex, then retract it quickly (Video 3). The resulting [Ca^2+^]_cyt_ signals in the stimulated cell are shown in Figure 3B. After recording, remove the sample as described above.

**Video 3.**
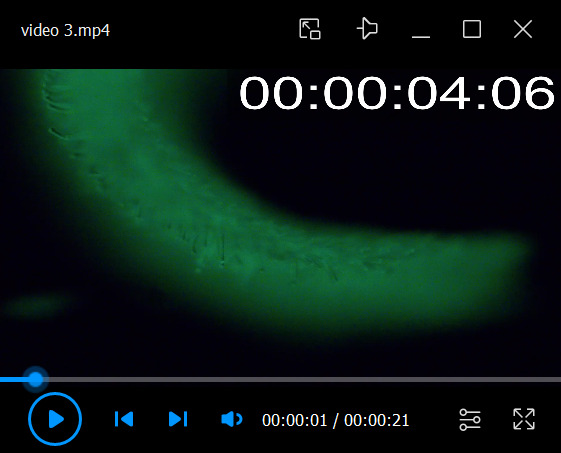
Side view of a papillar cell with a [Ca^2+^]_cyt_ wave after touch

## Data analysis

**Figure 4.**
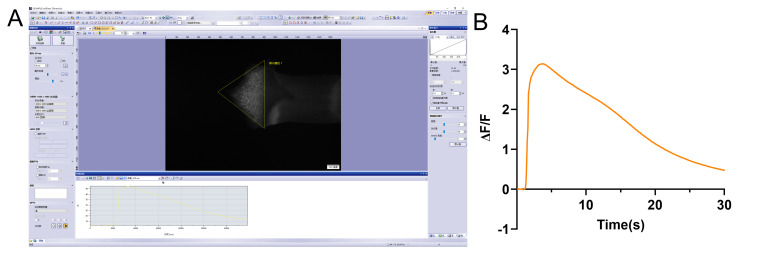
Analysis of touch-induced cytosolic Ca^2+^ dynamics. (A) Schematic depicting the region of interest (ROI, marked by yellow triangles) used for fluorescence intensity quantification in CellSens Dimension software. (B) Time-course of the relative average Ca^2+^ intensity (ΔF/F) across the entire stigma lobe following mechanical stimulation.

1. In CellSens Dimension software, convert the recorded video to 8-bit format. Define a region of interest (ROI) covering the entire touched lobe (Figure 4A) and measure the average fluorescence intensity within the ROI for each frame. The raw fluorescence intensity over time (Ft) is plotted (yellow line chart, Figure 4A).

2. Calculate the relative fluorescence intensity change (ΔF/F) using the equation: ΔF/F = (Ft - F0)/F0, where F0 is the average baseline fluorescence intensity from the 10 frames immediately preceding stimulation.

3. Analyze data from five stigmas as one experimental group using GraphPad Prism. Plot the mean ΔF/F from 10 frames pre-touch to the end of recording, using *points & connecting line with error bars* to generate the fitted curve shown in Figure 4B.

## Validation of protocol

This protocol has been used and validated in the following research article:

• Zhou et al. [11]. A mechanosensitive ion channel controls touch-triggered stigma movement through manipulation of calcium signature in *Torenia. Nat Commun.* 16(1): 6296. https://doi.org/10.1038/s41467-025-61770-6

## General notes and troubleshooting

1. Avoid selecting flowers in which both pairs of anthers have turned yellow. At this late developmental stage, stigma vitality is noticeably diminished, resulting in reduced movement range and attenuated calcium signaling upon touch.

2. The adhesive surface of the platform should be renewed after mounting approximately five stigmas. With repeated use, the tape's adhesion weakens, which can cause significant sample displacement during mechanical stimulation.

3. When mounting the style, ensure approximately 1 cm remains between the stigma lobes and the platform edge. If this clearance is too small, the stigma may adhere to the platform surface following stimulation.

4. The mechanical stimulation procedure with the tungsten needle must be performed swiftly. A slow or prolonged contact may obscure the observation of both the initial stigma response and calcium signals.

5. When imaging calcium dynamics in response to biotic or abiotic stresses (e.g., pathogen elicitors, salt, osmotic stress) alongside mechanically induced calcium signals, careful experimental design is required to avoid signal interference and ensure accurate interpretation. If both mechanical and stress-induced signals are to be recorded sequentially, allow sufficient recovery time between stimuli to avoid desensitization or overlap of calcium signals.
